# New insights into pyroptosis in pemphigus: from cellular structure to therapeutic targeting^☆^

**DOI:** 10.1016/j.abd.2024.07.015

**Published:** 2025-03-17

**Authors:** Jiazhen Chen, Zezhi He, Xiangnong Dai, Sifan Lin, Jiahui Liu, Xingdong Ye

**Affiliations:** a Guangzhou Dermatology Hospital, Guangzhou, Guangdong, China; b Guangzhou Medical University, Guangzhou, Guangdong, China

**Keywords:** Cell death, Inflammation, Pemphigus, Pyroptosis

## Abstract

**Background:**

Pemphigus is an autoimmune blistering disease where autoantibodies target desmoglein (Dsg) antigens on keratinocytes, triggering the p38 MAPK pathway, Dsg internalization, desmosomal dissolution, and keratinocyte apoptosis, are essential for blister formation. Recent research indicates keratinocyte pyroptosis may exacerbate acantholysis and delay wound healing. Current treatments, including corticosteroids and immunosuppressants, are effective but have significant side effects, such as prolonged wound healing and increased infection risk. Understanding these inflammatory processes is crucial for developing effective treatments for pemphigus.

**Methods:**

The authors conducted a comprehensive review of the literature, analyzing recent findings regarding the upregulation of pyroptosis-related proteins in pemphigus.

**Results:**

The present findings highlight a significant upregulation of pyroptosis-related proteins, which play a crucial role in the inflammatory response and blister formation characteristic of pemphigus. Key proteins such as cytokines IL-1β, IL-18, High Mobility Group Box-1 (HMGB1), and Parkin, along with NOD-like receptors and P2 × 7 receptors, were identified as pivotal in facilitating pyroptosis. The study also discusses potential therapeutic approaches targeting these proteins to modulate the disease pathway effectively.

**Study limitations:**

This study aimed to investigate the role of pyroptosis in the pathogenesis of pemphigus, focusing on its potential as a novel therapeutic target.

**Conclusions:**

Pyroptosis significantly contributes to the pathogenesis of pemphigus and presents a promising target for therapy. Targeting specific molecules involved in the pyroptosis pathway offers the potential for developing more precise and less toxic treatments, allowing the shift from traditional therapies towards targeted therapeutic strategies.

## Introduction

Pyroptosis is a kind of programmed cell death that occurs in a highly inflammatory environment. It is characterized by cell lysis, swelling, the creation of pores in the cell membrane, and the release of cell contents and pro-inflammatory mediators, all of which lead to cell death. Pyroptosis differs fundamentally from apoptosis and necrosis in cell form and mechanism, and it is involved in a variety of biological processes including immunological defense, inflammatory response, and disease progression. The role of the acantholysis pathway in pemphigus has recently attracted a lot of research interest.

Pemphigus is an autoimmune bullous skin condition characterized by a lack of attachment of the epidermis's Kerationcyte (KC) to the mucosa, resulting in blister formation. Pathogenic autoantibodies of the Desmoglein (Dsg) antigen activate various signaling pathways, which contribute to the illness. The pathogenesis of pemphigus is now based on four theories: the Dsg Compensation Hypothesis,^1^ the steric hindrance hypothesis,^2^ the multipathogenic theory/multiple hit theory, and the apoptotic lysis theory.^3^ Autoantibodies specifically target adhesion molecules between epidermal and mucosal KC, such as Dsg1 and Dsg3, causing cell disjunction and blistering. In addition to antibody-mediated effects, pemphigus causes alterations in intracellular signal transduction, such as endoplasmic reticulum stress response,^4^ cytoskeletal recombination,^5^ inflammatory mediator release,^6^ and apoptosis,^7^ and pyroptosis pathways.^8^

Understanding the clinical implications of pyroptosis in pemphigus is essential. Clinically, patients with pemphigus exhibit erosive lesions that are more difficult to heal compared to normal skin, and these erosions are prone to bacterial or viral infections. Investigating whether pyroptosis plays a significant role in these clinical manifestations is crucial. Pyroptosis, through its release of pro-inflammatory mediators, might exacerbate the inflammatory environment, leading to delayed healing and increased susceptibility to infections in pemphigus patients. Understanding the contribution of pyroptosis to these clinical outcomes could help in developing targeted therapies that improve wound healing and reduce infection rates, thereby enhancing the overall management of pemphigus. This study summarizes the scientific developments on pyroptosis in pemphigus.

## Activation mode of pyroptosis

Pyroptosis is caused by the activation of inflammasomes via two major pathways: classical and non-classical.^9^, ^10^ Toll-Like Receptors (TLR) or Nod-Like Receptors (NLR) on the cell surface recognize pathogenic or injury-related molecular patterns, which activate the pyroptotic cascade in the classical pathway. As a result, the inflammasome is generated, which is a protein complex that activates the pro-Caspase-1 precursor when it recognizes the presence of a pathogen or cell damage. Caspase-1 activation causes the cleavage of the Gasdermin-D (GSDMD) protein, resulting in the formation of GSDMD-N. GSDMD-N then causes pores in the cell membrane, resulting in changes in osmotic pressure, cell swelling, and, ultimately, membrane rupture.^11^ Caspase-1 promotes the production and release of inflammatory cytokines IL-1β and IL-18, triggering an inflammatory response.

Bacterial components such as Lipopolysaccharides (LPS) can activate Caspase-4/5/11 via a non-traditional method.^12^ Caspases 4, 5, and 11 activate the Pannexin-1 channel, which allows the cell to release ATP into the extracellular space. This activates the P2 × 7 channel on the cellular membrane, causing membrane pores to develop and the pyroptosis process to commence. Activated Pannexin-1 activates the NLRP3 inflammasome by releasing potassium ions, resulting in the creation and release of IL-1β and IL-18. This technique completes the release of inflammatory factors, accelerating the advancement of inflammation.^13^

## Expression of pyroptosis-related protein in pemphigus

### Secretory protein: Cytokine

**Figure 1:**
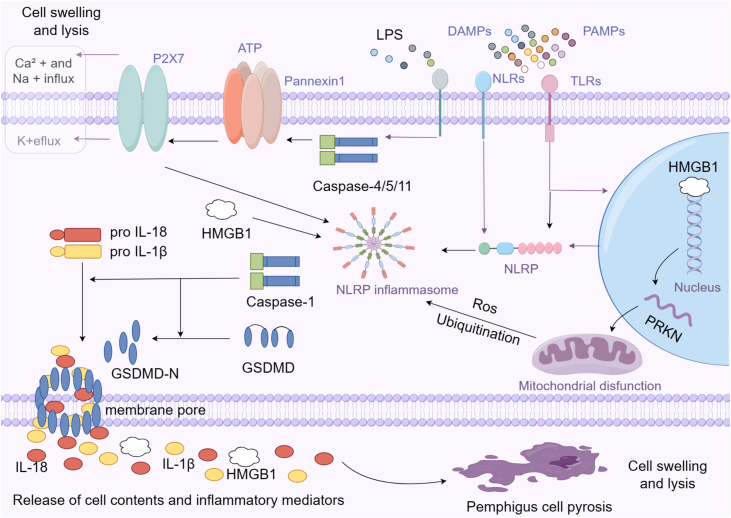
DAMPs and PAMPs are 'damage-associated molecular patterns' and 'pathogen-associated molecular patterns', respectively, that are recognized by pattern recognition receptors on cells, such as TLRs (Toll-Like Receptors) and NLRs (NOD-Like Receptors), to trigger and disseminate an immune response. Inflammasomes are multi-protein complexes that include NLRP proteins and are essential for innate immunity and the activation of inflammatory responses. Enzymes known as caspases are proteases that are crucial in inflammatory responses and programmed cell death. Caspase-1 and Caspase-4/5/11 are shown in the picture; they activate inflammasomes and cleave pro-inflammatory cytokines. GSDMD (Gasdermin-D): Upon cleavage by Caspase-1, GSDMD-N forms pores in the cell membrane, leading to the release of the cell's contents, including IL-1β, IL-18, and HMGB1. Upon stimulation by extracellular ATP, the P2 × 7 receptor opens and allows ions to flow, altering intracellular concentrations. This ionic change prompts the opening of Pannexin1 channels, facilitating the release of ATP and other inflammatory mediators. This ATP release and ion flux through P2 × 7 and Pannexin1 channels is a pivotal step in activating the inflammasome, leading to pyroptosis.

The IL-1 family's pro-inflammatory molecules, IL-1β and IL-18, play a crucial role in pyroptosis (Shown in the left part of Fig. 1). Research indicates that untreated patients with active pemphigus have high levels of IL-1β in their serum and tissues,^14^, ^15^, ^16^ but patients in remission have low levels. The increased expression of IL-1β may directly contribute to the inflammation and damage process in pemphigus. Tear IL-1β levels in patients with pemphigus were substantially elevated compared to those of healthy controls, as determined by Feng J et al. from pemphigus patients' tears.^17^ Huang et al. used microarray technology, GO enrichment, and KEGG pathway analysis to show that monocyte infiltration-related genes were highly expressed in pemphigus patients' skin lesions, with dense neutrophil infiltration, high expression of the IL-17 signaling pathway in skin lesions and peripheral blood monocytes, and peripheral blood monocytes responded abundantly to IL-1.^18^ IL-1 is a potent inducer of IL-17, which in turn recruits bone marrow cells that secrete IL-1,^19^ implying that there may be a positive feedback loop in pemphigus, with IL-1 amplifying pemphigus inflammation via IL-17-related signaling pathways. Hebert et al. discovered significantly elevated expression of IL-1β, IL-23p19, and IL-12p35 proinflammatory cytokine coding genes in autoreactive B-cells of pemphigus patients using quantitative polymerase chain reaction.^20^ In a study by Narbutt et al., pro-inflammatory cytokines (IL-1β, TNF-α, and IL-6) were found to be more expressed in cells incubated with pemphigus antibodies from active, remission, and healthy patients.^21^ Feliciani et al. created a mouse IL-1 gene knockout model and injected pemphigus antibodies. They discovered that animals with reduced IL-1β production had a lower incidence of pemphigus than the control group. This suggests that IL-1β promotes the occurrence or development of pemphigus. IL-1 knockout animals exhibited acantholysis, indicating that IL-1 may not be involved in the onset of pemphigus, but rather in the expansion of inflammation and injury.^22^ In a study by Kailash C et al.,^23^ serum IL-1 Receptor antagonist (Ra) levels increased in pemphigus patients in remission. This suggests that IL-1 Ra may inhibit the inflammatory effect of IL-1β and alleviate the disease. IL-1 inhibitors may also be effective in treating pemphigus.

### Structural proteins and signaling molecules: HMGB1

High Mobility Group Box-1 (HMGB1) is a non-histone chromatin-binding protein found in the nuclei of all mammalian cells. HMGB1 is a protein that serves multiple biological functions, including regulating DNA structure and function within the cell and acting as a pro-inflammatory signaling molecule outside the cell. In the cell, HMGB1 participates in DNA replication, repair, transcription, and chromatin remodeling. When cells are damaged or inflamed, HMGB1 can be released outside the cell, acting as an extracellular signaling molecule involved in inflammation, tissue repair, and immune regulation. The release of HMGB1 is regarded as an important marker of cell damage and death, and it has the ability to activate the immune system, induce and enhance an inflammatory response. Pyroptosis causes the release of cell contents, including HMGB1, which activates the inflammasome, leading to the production and release of inflammatory factors such as IL-1β and IL-18. HMGB1, on the other hand, can activate immune cells and direct the development of an immune response by binding to a variety of receptors, including receptor RAGE (a receptor for advanced glycation end products) and TLRs (Shown in the right part of Fig. 1). This effect promotes the formation and progression of inflammation during pyroptosis.^24^, ^25^ Li et al. investigated serum HMGB1 levels as well as tissue expression of HMGB1 and its receptor RAGE in pemphigus patients. They discovered that serum HMGB1 levels were significantly higher in pemphigus patients than in bullous pemphigoid patients and healthy control populations, and serum HMGB1 levels were significantly higher in pemphigus patients before treatment than after treatment. HMGB1 is abundant in the epidermal cytoplasm of pemphigus patients, whereas HMGB1 expression in healthy skin and bullous pemphigoid is almost entirely confined to the nucleus.^26^ The change in HMGB1 level is closely related to disease activity and treatment response, implying that HMGB1 may be an important part of the pathological mechanism of pemphigus, and that HMGB1 may be both a biomarker of disease activity and an indicator of treatment outcome.

### Mitochondria-related functional proteins: Parkin

Parkin, encoded by the PRKN gene, is expressed in both the cytoplasm and the nucleus and plays a role in protein degradation and mitochondrial function maintenance. Parkinin regulates autophagy, which helps to maintain mitochondrial homeostasis. Dysfunctional mitochondria can produce Damage-Associated Molecular Patterns (DAMPs) and Reactive Oxygen Species (ROS), which are known to activate the inflammasome and cause pyroptosis. PRKN mutations or dysfunctions predispose to inflammation and create pyrogenic environments. Parkin proteins, on the other hand, are involved in the regulation of protein degradation in cells via the Ubiquitin-Proteasome System (UPS) and the autophagy pathway (Shown in the right part of Fig. 1). Parkin protein can ubiquitinate a variety of substrates, recognizing and promoting the degradation of markedly damaged or over-accumulated proteins, thereby influencing inflammatory signaling pathways.^27^, ^28^ Bumiller-Bini et al. used microarray hybridization and multivariate logistic regression to systematically study the allele and genotype frequencies encoding all 12 mature cell death cascades in 227 patients with pemphigus foliaceous and 194 controls. The pyrogenic cell death gene PRKN was discovered to be a protective factor for pemphigus foliaceus.^29^ This suggests that the PRKN gene may play a protective role in pemphigus patients, as its normal function promotes mitochondrial health, reduces inflammatory response, and inhibits the scorch-death process. This discovery sheds new light on the pathogenesis of pemphigus.

### Proteins with roles in responding to and modulating inflammation: NLR

Nucleotide-binding oligomerization domain-like receptors (NOD-like receptors) (also known as NLR) are a group of proteins with over 20 subtypes. These include NOD1, NOD2, NLRP1, NLRP3, and NLRC4 (also known as IPAF), which are intracellular Pattern Recognition Receptors (PRRs). These receptors activate inflammatory responses and cell death pathways in host defense mechanisms by recognizing endogenous molecules, bacteria, viruses, and toxic foreign bodies in the cytoplasm.^30^, ^31^ Furthermore, NLR can interact with other signaling proteins to form an inflammasome, which activates downstream inflammatory signaling pathways. The NLR protein family's inflammatory bodies activate Caspase, resulting in the maturation of Caspase-1 substrates like IL-1β and IL-18, which initiate immune and inflammatory responses. Shamsabadi et al. used real-time polymerase chain reaction to discover that NLRP1 and IPAF mRNA levels in patients with active pemphigus were significantly higher than in healthy controls.^15^ The upregulation of NLRP1 and IPAF in pemphigus patients may indicate over-activation of these inflammatory bodies (Shown in the central part of Fig. 1). Inflammatory mediators like IL-1β and IL-18 may also contribute to skin inflammation and damage. These findings indicate that the NLR protein family which is related to pyroptosis may play an important role in the pathogenesis of pemphigus.

### Membrane channel protein: P2XR

Membrane channel Protein P2X Receptor (P2XR) is a class of ion channel receptors made up of seven subtypes, P2 × 1–P2 × 7, that belong to the ATP-dependent ion channel on the cell surface. Extracellular ATP activates these receptors, causing Calcium ions (Ca² ^+^) and sodium ions (Na^+^) to enter the cell and potassium ions (K^+^) to leave. P2X receptors are more closely related to pyroptosis. Sustained activation of P2 × 7 causes extracellular ATP release, K + ion efflux, inflammasome activation, and Caspase-1 activation, which promotes IL-1β and IL-18 secretion. Furthermore, activating the P2 × 7 receptor increases membrane permeability and promotes the release of cell contents, such as pro-inflammatory factors^32^, ^33^ (Shown in the upper area of Fig. 1). These factors may cause or exacerbate pyroptosis. Malheiros et al. compared the genome-wide gene expression profiles of peripheral CD4+ T-cells between different subgroups of pemphigus foliaceus patients and healthy individuals, discovering that the P2XR gene was highly expressed in untreated pemphigus patients.^34^ It is also proposed that ATP and P2X receptors play important roles in tissue inflammation and cell pyroptosis in pemphigus.

### Relationship of pyroptosis and acantholysis

**Figure 2:**
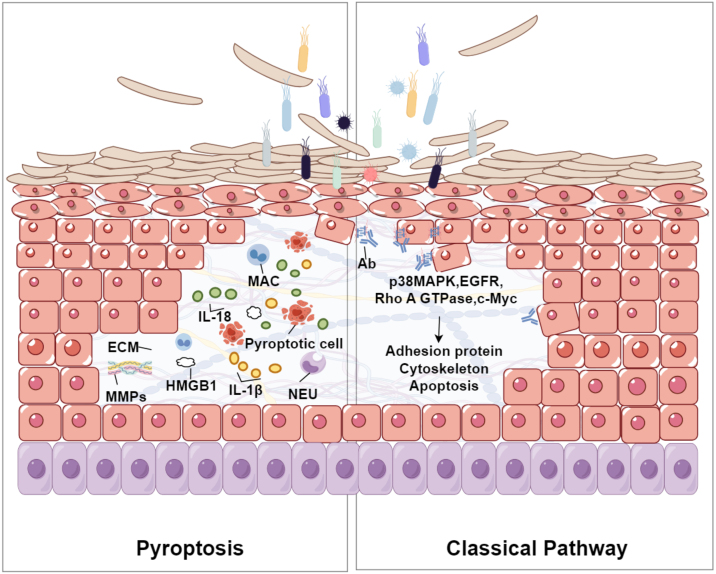
The relationship between pyroptosis and acantholysis within the epidermis. Pyroptotic cells, characterized by the formation of Gasdermin-mediated pores, release pro-inflammatory cytokines such as IL-1β and IL-18. These cytokines attract Neutrophils (NEU) and Macrophages (MAC), intensifying the inflammatory environment. The resultant oxidative stress and production of Matrix Metalloproteinases (MMPs) may degrade the Extracellular Matrix (ECM), weakening the structural integrity of the epidermis and leading to the detachment of keratinocytes, a process known as acantholysis. This figure illustrates the complex interplay between cellular death, inflammation, and tissue disruption in the pathogenesis of blistering skin disorders. The right part of the figure shows the classical pathway of acantholysis in pemphigus. Bacterial interaction triggers antibody production, activating signaling molecules (p38 MAPK, EGFR, Rho A GTPase, c-Myc), which affect the cytoskeleton and adhesion proteins, leading to cell detachment and apoptosis.

The relationship between pyroptosis and acantholysis may primarily revolves around the inflammatory response triggered by pyroptosis and its detrimental impact on skin tissue integrity. Pyroptosis is a form of programmed cell death that involves inflammasome activation, pore formation, and subsequent cell lysis. It is associated with various inflammatory and autoimmune conditions. Acantholysis refers to the loss of adhesion between keratinocytes in the epidermis, leading to blistering skin disorders. During pyroptosis, Gasdermin proteins form pores in the cell membrane, causing cell swelling and rupture. This process triggers the release of substantial quantities of pro-inflammatory cytokines, such as IL-1β and IL-18, fostering an inflammatory environment that can exacerbate skin conditions. These cytokines promote various immunological responses, including leukocyte migration and the production of other inflammatory mediators, which may further aggravate acantholysis^35^ (Shown in Fig. 2). Pyroptosis is commonly activated in response to intracellular pathogens, resulting in skin inflammation. In this process, oxidative stress induces mitochondrial dysfunction and the generation of Reactive Oxygen Species (ROS), which exacerbates cellular damage and inflammation. Additionally, Nitric Oxide (NO) released during pyroptosis enhances the production of Matrix Metalloproteinases (MMPs) and inflammatory cytokines, disrupting the Extracellular Matrix (ECM) integrity and leading to skin lesions.^36^, ^37^, ^38^ In chronic inflammatory skin diseases such as eczema, seborrheic dermatitis, or psoriasis, the exacerbation of symptoms can be attributed to the pro-inflammatory cytokines released during pyroptosis.^35^, ^39^, ^40^ Persistent inflammation compromises the structural integrity of the skin, rendering it more susceptible to acantholytic changes. Elevated levels of inflammatory cytokines, including IL-18 and IL-1β, have been documented in several skin disorders, suggesting their potential role in linking pyroptosis to acantholysis. These sources provide a comprehensive understanding of how pyroptosis contributes to the pathogenesis of acantholysis by releasing inflammatory mediators, causing oxidative stress, and resulting in tissue damage. This information offers valuable insights into potential therapeutic targets for treating related skin conditions.

### Treating pemphigus with pyroptosis-related targets

Exploring the role of pyroptosis in pemphigus pathogenesis and its therapeutic implications requires digging deeper into Caspase activation and how Caspase inhibitors, particularly those targeting Caspase-1, can help treat the disease. Classically, autoantibodies that disrupt keratinocyte adhesion cause acantholysis and blisters in pemphigus. A recent study reveals that cell death processes, notably pyroptosis, contribute to its pathogenesis. Pyroptosis is triggered by Caspase family members like Caspase-1, Caspase-4, Caspase-5 and Caspase-11, which recognize intracellular pathogens or damage-related molecular patterns. Numerous animal, cellular, and human studies have demonstrated that Caspase activation is harmful in experimental pemphigus, and pancaspase inhibitors can block or diminish acantholysis and blister formation in pemphigus in vitro and in vivo, respectively.^41^, ^42^, ^43^, ^44^, ^45^ Wang et al. used Trypan blue in vivo staining to evaluate cell mortality and found that YVAD-CHO, a Caspase-1 inhibitor, could decrease PV-IG-induced keratinocyte death and tissue acantholysis.^46^ The effect of pyroptosis-specific Caspase-1 inhibitors supports the idea of pyroptosis in pemphigus pathogenesis and highlights the potential for targeting specific Caspases in pemphigus treatment.

## Conclusion and prospects

Pemphigus refers to a class of autoimmune bullous skin illnesses in which intercellular adhesion is lost due to the generation of autoimmune antibodies. Recent research has revealed that innate immunity, including the recruitment of innate immune cells, the release of inflammatory mediators, and the activation of the complement system, also plays a significant role in the pathological process of pemphigus. Pemphigus is primarily linked to abnormal adaptive immunity, particularly autoantibodies produced by B-cells. A type of planned cell death called pyroptosis is closely linked to inflammation, which is a result of both innate immune system and inflammatory body activation. In the development and progression of pemphigus, NLRP inflammatory bodies, Caspase, IL-1 and IL-18, PRKN, and P2X are considered to be implicated. In-depth research is still lacking to determine whether pyroptosis is linked to pemphigus skin infection and refractory erosion. This includes the significance of Gasdermin D, the key pyroptosis protein, being expressed in pemphigus and the mechanism by which pyroptosis-related molecules activate pyroptosis through a particular signaling pathway. It is also unclear whether pyroptosis broadens the inflammatory range of pemphigus. In vitro and in vivo, inhibitors of pancaspase, a pyroptosis-related molecule, prevent or lessen acantholysis and blister development in pemphigus. It has also been demonstrated that Caspase-1 inhibitors, an important pyroptosis enzyme, are useful in preventing the creation of vesicles; however, further experimental confirmation is required. More research is required to determine the potential of IL-1 receptor antagonists in the management of pemphigus.

## Financial support

The Guangzhou Science and Technology Bureau (NO. 2023A03J0946).

## Authors’ contributions

Jiazhen Chen: Conducted a comprehensive literature review, wrote the initial draft of the manuscript and created the figures.

Zezhi He: Created the figures and provided the necessary resources.

Xiangnong Dai: Contributed to the critical review and editing of the manuscript.

Sifan Lin: Contributed to the critical review and editing of the manuscript.

Jiahui Liu: Supervised the research and provided the necessary resources.

Xingdong Ye: Conceived the original idea and designed the framework of the study.

## Conflicts of interest

None declared.
